# Supplementary material for: Reaction time coupling in a joint stimulus-response task: A matter of functional actions or likable agents?
Source: PLoS One. 2022 Jul 12;17(7):e0271164. doi: 10.1371/journal.pone.0271164 (PMC9275686; doi:10.1371/journal.pone.0271164)
Supplement: S1 Fig — Linear regression fits to reaction times per trial of participants (y-axis) as a function of reaction times of sagents (x-axis) per experiment (A-C). Note that the distribution of reaction times of agents varied across individuals due to random sampling of reaction times. (DOCX) [file pone.0271164.s001.docx]

**
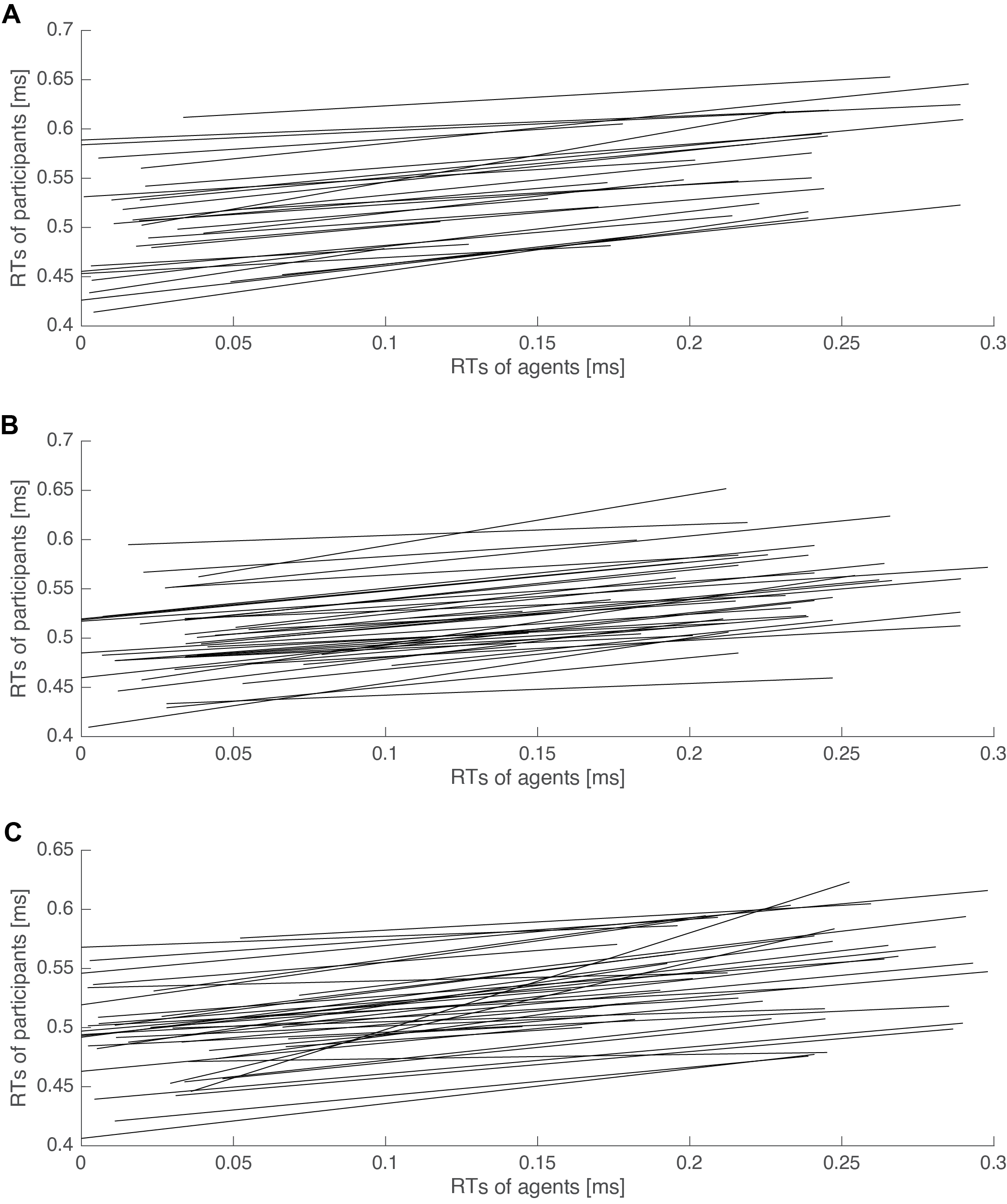
**

**S1 Fig. Reaction times of participants as a function of reaction times of agents per experiment.** Linear regression fits to reaction times per trial of participants (y-axis) as a function of reaction times of agents (x-axis) per experiment (A-C). Note that the distribution of reaction times of agents varied across individuals due to random sampling of reaction times.
