# Supplementary material for: Reaction time coupling in a joint stimulus-response task: A matter of functional actions or likable agents?
Source: PLoS One. 2022 Jul 12;17(7):e0271164. doi: 10.1371/journal.pone.0271164 (PMC9275686; doi:10.1371/journal.pone.0271164)
Supplement: S2 Fig — Mean and standard errors across participants of correlations of reaction times between participants and agents (y-axis) per agent type (x-axis) and per experiment (A-C). Func = Functional (correct choices), Dysfunc = Dysfunctional (incorrect choices), Neu = Neutral, Like = Likable (cooperative), Dislike = Dislikable (uncooperative). (DOCX) [file pone.0271164.s002.docx]

**
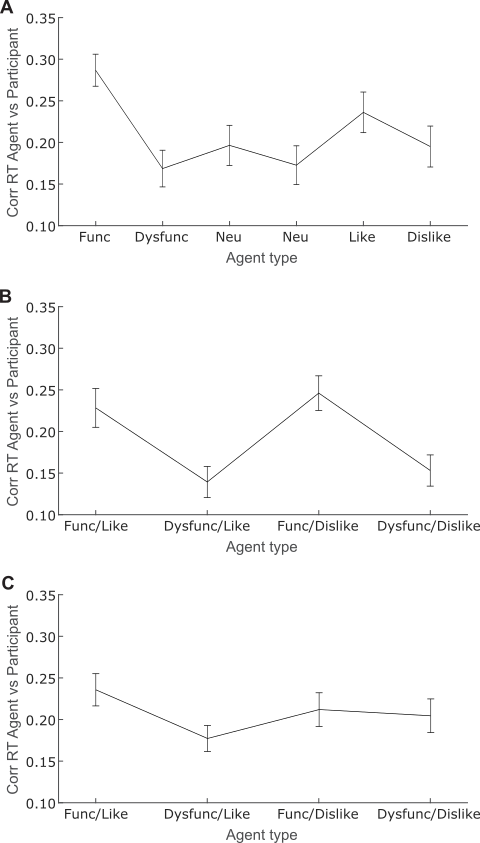
**

**S2 Fig. Reaction times per agent type per experiment.** Mean and standard errors across participants of correlations of reaction times between participants and agents (y-axis) per agent type (x-axis) and per experiment (A-C). Func = Functional (correct choices), Dysfunc = Dysfunctional (incorrect choices), Neu = Neutral, Like = Likable (cooperative), Dislike = Dislikable (uncooperative).
