# Supplementary material for: Reaction time coupling in a joint stimulus-response task: A matter of functional actions or likable agents?
Source: PLoS One. 2022 Jul 12;17(7):e0271164. doi: 10.1371/journal.pone.0271164 (PMC9275686; doi:10.1371/journal.pone.0271164)
Supplement: S1 Table — Based on their choice to defect or cooperate, the participant and agent could earn or lose points. In experiment 2 & 3 participants could earn or lose money (numbers are in cents rather than points). (DOCX) [file pone.0271164.s004.docx]

**S1 Table.** *The pay-off matrix of the PD in experiment 1 and experiment 2 & 3. Based on their choice to defect or cooperate, the participant and agent could earn or lose points. In experiment 2 & 3 participants could earn or lose money (numbers are in cents rather than points).*

| Experiment 1 | Agent | | |
| --- | --- | --- | --- |
| Participant |  | Cooperate | Defect |
|  | Cooperate | +20, +20 | -20, +10 |
|  | Defect | +10, -20 | -20, -20 |
|  |  |  |  |
| Experiment 2 & 3 | Agent | | |
| Participant |  | Cooperate | Defect |
|  | Cooperate | 5, 5 | 0, 10 |
|  | Defect | 0, 0 | 2, 2 |
