# Supplementary material for: Reaction time coupling in a joint stimulus-response task: A matter of functional actions or likable agents?
Source: PLoS One. 2022 Jul 12;17(7):e0271164. doi: 10.1371/journal.pone.0271164 (PMC9275686; doi:10.1371/journal.pone.0271164)
Supplement: S2 Table — The questions had to be answered on a continuous dimension scale of 0 to 100. (DOCX) [file pone.0271164.s005.docx]

**S2 Table.** *Questions to evaluate to what degree the behavioral manipulations of the agents were noticed by the participants. The questions had to be answered on a continuous dimension scale of 0 to 100.*

| 1. | How likeable was the agent during the prisoner's dilemma game? |
| --- | --- |
| 2. | How cooperative was the agent during the prisoner's dilemma game? |
| 3. | How functional/well did the agent play the color action game? |
| 4. | How functional/well did the agent made his/her choices in the color action game? |
