# Supplementary material for: Reaction time coupling in a joint stimulus-response task: A matter of functional actions or likable agents?
Source: PLoS One. 2022 Jul 12;17(7):e0271164. doi: 10.1371/journal.pone.0271164 (PMC9275686; doi:10.1371/journal.pone.0271164)
Supplement: S3 Table — (DOCX) [file pone.0271164.s006.docx]

**S3 Table.**  *Experiment 1 one-way ANOVA results (F-statistic, p-value, partial eta squared) on subjective ratings, with the predicting factors of agent type per rating type.*

| **Ratings** | **df** | ***F, p,*  *η*_p_^2^** |
| --- | --- | --- |
| Likability | 4, 116 | *18.44,* *<.001, .39* |
| Functionality | 3.4, 99.7 | *7.54,* *<.001, .21* |
