# Supplementary material for: Reaction time coupling in a joint stimulus-response task: A matter of functional actions or likable agents?
Source: PLoS One. 2022 Jul 12;17(7):e0271164. doi: 10.1371/journal.pone.0271164 (PMC9275686; doi:10.1371/journal.pone.0271164)
Supplement: S4 Table — (DOCX) [file pone.0271164.s007.docx]

**S4 Table.** *Experiment 1 post-hoc t-test results (t-value, p-value, Cohen’*s d*) on subjective ratings compared across agent types (dof = 29; number represent comma-separated t-statistics and p-value)*.

|  | **Likability rating (*t, p, d*)** |  |  |  |  |  |
| --- | --- | --- | --- | --- | --- | --- |
|  | **Like. Pos.** | **Like. Neu.** |  |  | **Func. Pos.** | **Func. Neu.** |
| **Like. Neu.** | *3.99, < .001, 0.72* |  |  | **Func. Neu.** | *0.30, .766, 0.05* |  |
| **Like. Neg.** | *6.72, < .001, 1.23* | *5.14, <.001* |  | **Func. Neg.** | *0.58, .564, 0.11* | *0.82, .416, 0.15* |
|  | **Functionality rating** |  |  |  |  |  |
|  | **Func. Pos.** | **Func. Neu.** |  |  | **Like. Pos.** | **Like. Neu.** |
| **Func. Neu.** | *0.09, .925, 0.02* |  |  | **Like. Neu.** | *0.34, .737, 0.06* |  |
| **Func. Neg.** | *7.62, <.001, 1.39* | *4.84, .002, 0.88* |  | **Like. Neg.** | *0.31, .758, 0.06* | *.60, .556, 0.11* |
