# Supplementary material for: Reaction time coupling in a joint stimulus-response task: A matter of functional actions or likable agents?
Source: PLoS One. 2022 Jul 12;17(7):e0271164. doi: 10.1371/journal.pone.0271164 (PMC9275686; doi:10.1371/journal.pone.0271164)
Supplement: S5 Table — (DOCX) [file pone.0271164.s008.docx]

**S5 Table**. *Experiment 2 two-way repeated measures ANOVA results (F-statistic, p-value, partial eta squared) on subjective ratings with agent functionality (dof = 1, 41) and agent likability as predictors (dof = 0.9, 36.4).*

| **Ratings** | **Main effect** | ***F, p, η*_p_^2^** |
| --- | --- | --- |
| Likability | Functionality | *6.84, .012, 0.12* |
|  | Likability | *133.31,* *<.001, 4.97* |
|  | Functionality * Likability | *0.12,* *.731, <0.01* |
|  |  |  |
| Functionality | Functionality | *58.69,* *<.001, 1.17* |
|  | Likability | *0.03,* *.860, <0.01* |
|  | Functionality * Likability | *3.62,* .*064, 0.09* |
