# Supplementary material for: Reaction time coupling in a joint stimulus-response task: A matter of functional actions or likable agents?
Source: PLoS One. 2022 Jul 12;17(7):e0271164. doi: 10.1371/journal.pone.0271164 (PMC9275686; doi:10.1371/journal.pone.0271164)
Supplement: S6 Table — Abbreviations: L+ F+: likable, functional; L+ F-: likable, dysfunctional; L- F+: dislikable, functional; L- F-: dislikable, dysfunctional. (DOCX) [file pone.0271164.s009.docx]

**S6 Table**. *Experiment 2* *post-hoc t-test results (t-value, p-value, Cohen’s d) on subjective ratings compared across agent types (def = 41). Abbreviations*: *L+ F+: likable, functional; L+ F-: likable, dysfunctional; L- F+: dislikable, functional; L- F-: dislikable, dysfunctional.*

| **Likability (*t, p, d*)** | | | |
| --- | --- | --- | --- |
|  | **L+F+** | **L+F-** | **L-F+** |
| **L+ F-** | *1.52, .135, 0.24* |  |  |
| **L- F+** | *8.38, < .001, 1.29* | *6.86, <.001, 1.06* |  |
| **L- F-** | *13.93, <.001, 2.15* | *9.65, <.001, 1.49* | *1.84, .070, 0.28* |
| **Functionality** | | | |
|  | **L+F+** | **L+F-** | **L-F+** |
| **L+ F-** | *3.73, <.001, 0.58* |  |  |
| **L- F+** | *-1.19, .241, 0.18* | *-4.71, <.001, 0.73* |  |
| **L- F-** | *4.86, <.001, 0.75* | *1.33, .190, 0.21* | *6.53, <.001, 1.01* |
