# Supplementary material for: Reaction time coupling in a joint stimulus-response task: A matter of functional actions or likable agents?
Source: PLoS One. 2022 Jul 12;17(7):e0271164. doi: 10.1371/journal.pone.0271164 (PMC9275686; doi:10.1371/journal.pone.0271164)
Supplement: S7 Table — (DOCX) [file pone.0271164.s010.docx]

**S7 Table.** *Experiment 3* *two-way repeated measures ANOVA results (F-statistic, p-value) on subjective ratings with agent functionality and agent likability as predictors (dof = 1, 41).*

| **Ratings** | **Main effect** | ***F, p, η*_p_^2^** |
| --- | --- | --- |
| Likability | Functionality | *0.69, 0.413, 0.01* |
|  | Likability | *162.02, <.001, 2.73* |
|  | Functionality* Likability | *0, 0.953, <0.01* |
|  |  |  |
| Functionality | Functionality | *95.84, <.001, 1.11* |
|  | Likability | *0.06, 0.808, <0.01* |
|  | Functionality* Likability | *1.12, 0.297, 0.03* |
