# Supplementary material for: Reaction time coupling in a joint stimulus-response task: A matter of functional actions or likable agents?
Source: PLoS One. 2022 Jul 12;17(7):e0271164. doi: 10.1371/journal.pone.0271164 (PMC9275686; doi:10.1371/journal.pone.0271164)
Supplement: S8 Table — Abbreviations: L+ F+: likable, functional; L+ F-: likable, dysfunctional; L- F+: dislikable, functional; L- F-: dislikable, dysfunctional. (DOCX) [file pone.0271164.s011.docx]

**S8 Table.** *Experiment 3* *post-hoc t-test results (t-value, p-value, Cohen’s d) on subjective ratings compared across agent types (dof = 41). Abbreviations*: *L+ F+: likable, functional; L+ F-: likable, dysfunctional; L- F+: dislikable, functional; L- F-: dislikable, dysfunctional.*

| **Likability (*t, p, d*)** | | | |
| --- | --- | --- | --- |
|  | **L+F+** | **L+ F-** | **L-F+** |
| **L+ F-** | *-0.54, 0.594, 0.08* |  |  |
| **L- F+** | *8.23, <.001, 1.27* | *9.49, <.001, 1.46* |  |
| **L- F-** | *7.89, <.001, 1.22* | *8.043, <.001, 1.24* | *-0.57, 0.573, 0.09* |
| **Functionality** | | | |
|  | **L+F+** | **L+ F-** | **L-F+** |
| **L+ F-** | *4.60, <.001, 0.71* |  |  |
| **L- F+** | *-0.96, 0.345, 0.15* | *-6.17, <.001, 0.95* |  |
| **L- F-** | *5.68, <.001, 0.88* | *0.613, 0.543, 0.09* | *6.56, <.001, 1.01* |
