# Supplementary material for: Reaction time coupling in a joint stimulus-response task: A matter of functional actions or likable agents?
Source: PLoS One. 2022 Jul 12;17(7):e0271164. doi: 10.1371/journal.pone.0271164 (PMC9275686; doi:10.1371/journal.pone.0271164)
Supplement: Attachment — Submitted filename: Reviews_MN_21-9.docx [file pone.0271164.s012.docx]

Reviewers' comments:

Reviewer #1: Summary

The authors investigated whether coupling strength between individuals (measured via the variation in correlation in response times) is impacted by the perceived likability of the partner, or their perceived competence in unintentional coordination. Manipulations to like-ability was done using the prisoner’s dilemma game, and manipulations to the competency of the partner (or “functionality”) was manipulated by their congruency of responses to the CA game (Experiments 1 and 2) or their competency to answer questions on a quiz (Experiment 3). To confirm the manipulations had the desired effect, participants also answered a set of questions rating the agents’ likeability and competency (functionality).

Across the three experiments, the researchers found that variation in the competence of the agent in all three experiments impacted the degree of coupling in response times between the participant and the agent in the CA game. Variation in likeability did not change the correlation. Thus, the researchers demonstrate that making the competency of the partner salient impacts the degree of perceptual-motor coupling to that partner.

General Comments/Questions

**Comment 1.1:** The authors have drawn from speeded response paradigms to motivate their research study. There is a line of research from researchers in the ‘coordination dynamics’ literature that has investigated the role of pattern stability and social factors on interpersonal coordination whose inclusion would benefit this paper. In particular, the work of Lynden Miles (University of Western Australia) would be of relevance here. Miles has investigated the effects of rapport and group membership, for example, on the degree of coupling between people during rhythmic coordination tasks. In their work, social variables has an influence on the amount of coordination that is observed between people.

***Reply 1.1:*** *We thank the reviewer for the nice summary of our study. We also thank the reviewer for pointing out the work of Lynden Miles. We now cite this work at multiple places in the introduction and discussion (see line 86, 90, 127, and 551-553).*

**Comment 1.2:** I agree with the authors that despite possible limitations in their study, the fact that manipulations to competence, but not likability, had an impact on response time correlations is a novel finding. These findings support the view that coupling between one’s own action and what is observed is influenced related to their predictability.

One major challenge I have with the presentation of the study and the interpretation of the findings is how quickly the researchers are to conclude that the findings are tied to social imitation and mimicry, as opposed to a potentially non-social explanation. This cannot be fully determined as there was no “non-social” version of the task presented to participants. For example, would the researchers expect the same findings in re: to functionality if there was no “second person” on the experiment? For example, would the presentation of divergent answers in the quiz produce similar findings in Experiment 3? The conclusion I draw from the Experiments is that predictability of the task environment modulates attention by participants to complete the task. The researchers should acknowledge that this is a possibility and a limitation to their work, or provide a stronger argument why this is a social effect.

***Reply 1.2:*** *We thank the reviewer for pinpointing the relevance of our study. We agree that the task may not qualify as a typical social task. However, it is the behavior of the agents that affect the feelings of the participant that we denote as a social effect. These feelings may indeed modulate the degree of attention given to the agent’s actions and the RT coupling therewith. We now stress this possibility in the discussion (see line 563-567):*

*Such knowledge may set the action schemes of observers, like the possibility that, as agents performed more and more correct actions, participants may have gradually strengthened their focus of attention for the actions of agents. It is thus possible that not social contexts but factors that control attention affect perception-action coupling in specifically reaction time and stimulus-response tasks.*

Specific Comments

Comment 1.3 The title is loaded with terms I think is inappropriate given the task and findings. “Likeability” might be more appropriate than “Social Bonding” and I would argue that “learning” is an inappropriate word here and something along the lines of “modulation of attention” would be more appropriate.

Reply 1.3: We agree with the reviewer that the terms social bonding and learning were perhaps too broad and not necessarily relate to the factors that we manipulated in the experiments. As such, we changed the previous title “Perception-action pairing in a stimulus-response compatibility task: a matter of social bonding or learning?” to a title with better specifications: “Reaction time coupling in a joint stimulus-response task: a matter of functional actions or likeable agents?”. We did not incorporate the term attention in the title as we did not directly manipulate attention, and because this is, similar to the learning account of imitation, an interesting though only one of multiple underlying mechanisms that can explain the observed RT coupling. Note that we do bring forward attention as a potentially underlying mechanism of the here observed effects in the discussion section (see **reply 1.2**).

comment 1.4. For the color action (CA) game, were participants told that the videos that played were from the participant they were completing the task with, or pre-recorded videos? If participants are told they are pre-recorded – are they told they are of their partner?

Reply 1.4: We now clarify this in the methods of experiment 1 by emphasizing that we told participants that they would see live video footage of their partner (i.e., agent):

Line 229-230: We told the participants that this was a live video stream of the partner that they played with in the preceding PD game.

Comment 1.5. For the CA game in Method, clarify what is a congruent and incongruent action.

Reply 1.5: We now clarify this in the methods as follows:

Line 222-228: The CA game, essentially consisting of a virtual form of a joint compatibility task, incorporated cue-congruent and cue-incongruent actions. Cue-congruent trials consisted of trials in which the chosen color by the agent was in line with the shown color of the cue. In cue-incongruent trials the color choice of the agent differed from the cue’s color. Note, however, that any effects related to compatibility fall out of scope of the current study. The main purpose of the correct and incorrect agent trials in the CA game was to manipulate action functionality but not to study compatibility effects.

Comment 1.6. Lines 284-286. I’m confused what this statistical test is compared to the t-tests in the preceding sentences (lines 280-282). Also, results for Exp. 2 and 3 were generally easy to follow but was a little more of a challenge for Exp. 1. May I suggest using sub-headings that provide a predictable structure for all 3 experiments.

Reply 1.6. We apologies for the confusion. We clarified the purpose of the statistical t-test. This t-test compares whether the difference in RT correlation between the likability conditions (likable-not likable) differed from (i.e., was smaller than) the difference in RT correlation between the functionality conditions (functional-not functional). A significant difference means that the effect of functionality on RT correlation is larger than the effect of likability. Here are examples of the changes:

Line 338-341: To investigate the differences in degree of coupling between the likable versus functional agent characteristic, the effect of functional versus dysfunctional agents was compared with the effect of the likable versus non-likable agent.

Additionally, we thank the reviewer for suggesting the use of sub-headings. The use of sub-headings made the results sections much more structured and clearer.

Comment 1.7: Line 358. No Text under “Introduction” subheading. Unsure if this was intentional.

Reply 1.7. We accidentally omitted the text. We have added the following text under the introduction header of experiment 2:

Line 421-422: Experiment 2 builds on experiment 1, with the aim to provoke a higher social involvement of the participant by strengthening the effect of the likability manipulation.

Comment 1.8. It is unclear in the text what was the nature of the pre-recorded videos in the CA game for Experiment 3. Were all the videos congruent-only? (I assumed it is).

Reply 1.8. We thank the reviewer for pointing out that this was not sufficiently explained. We have added more text to explain the congruency/incongruency of the videos in experiment 3:

Line 505-506: Therefore, all the agents scored on average during the CA game by setting the number of congruent trials at 50 percent (40/80 trials).

Comment 1.9. In re: Discussion. Similar to my concerns with the title, I think potentially loaded terms are being used to explain the findings. For example attributing “trustworthiness” to the second person to explain the coupling correlations might be too much attribution for something that may be due to modulation of attention due to the predictability of responses in the task.

Reply 1.9. We agree with the reviewer that the paragraph discussing trustworthiness was too speculative. We have therefore removed this paragraph.

Comment 1.10. Related to my concerns above, my thoughts are motivated by similar concerns raised by Thomas Dolk’s work on referential coding in the joint simon task – where, certain “social” effects can be explained non-socially. I have not read the Dolk 2014 article the researchers cite, but I’m curious if that makes similar conclusions. This may also explain why the likability manipulation did not have an impact on the findings – because the explanation of the findings may be non-social.

Reply 1.10: Studies described by the review of Dolk et al. (2014), including a study by themselves (Dolk et al., 2011), indeed provide evidence that congruency effects, typically studied with the joint Simon task, can be best explained as the result of a non-social phenomenon, mostly because the mere believe that the task is performed together with another person (sitting in another room), causes changes in reaction times. We now discuss this work in the context of the lacking effect of likability (see line 559-567).

Reviewer #2: Content
The paper’s aim was to find out, as Author write: “To what degree the functionality and likability of agents shape reaction time synchronization”. The synchronization was measured as a correlation of reaction times in a congruency color task (“the same game”) played by an alleged confederate “several milliseconds in advance”.
Agents varied in likeability and “functionality”, which was evoked by manipulating their cooperativeness in prisoner’s dilemma and correctness of choices in the “preceding” games. The correlations were affected more by “functionality” of the agents than their likeability. Two more experiments were conducted to check if a stronger likeability manipulation would affect the correlations (it did not), and to manipulate the skillfulness (“functionality”) of the agents in a different way than with the same task on which the correlations were measured.
Author conclude that a stronger correlation of the RTs with “functional” agents means that it is the “potential to benefit of learning from others” that drives perception and action coupling.

Evaluation
**Comment 2.1:** The question posed by the Authors (if the timings of actions become more similar when the partner is perceived as cooperative and competent) is potentially interesting. The manipulations seemed to have the desired effect as shown by the ratings. A potentially interesting result is a weak but significant correlation of the reaction times in general.
However, I have strong reservations as to the other aspects of the paper. I had difficulty to ascertain from the descriptions if the design of the study indeed allowed to address the research questions, and if the questions were posed clearly on the theoretical level.

Reply 2.1: We thank the reviewer for the concise summary of our study. We have considerably improved the descriptions of the designs of the study (see other replies) and we are confident that the improved information now allows readers to fully understand the research questions. Most of the raised issues related to questions about congruency/compatibility analyses and unclear terminology. We now more clearly explain that the analyses related to congruency/compatibility are out of scope of the current study and we modified several terms throughout the manuscript to better specify the type of behavior that we were interested in.

Comment 2.2. Theory and formulating research question and hypotheses:
Some of the terms in the paper are used in a non-standard way. For example, the first sentence of the abstract says: “Being influenced by others’ behavior is a deep-rooted mechanism.” Is this really a mechanism, or we are rather seeking mechanisms underlying such a phenomenon?

Reply 2.2 We are glad the reviewer noticed this unusual wording, and we changed this and several other sentences throughout the manuscript.

Comment 2.3. I am not sure why the phenomenon is called “perception-action” pairing. Isn’t it rather matching of the perceived timings of actions of the collaborator with the timings of own actions? I will assume the Author’s expression is a shortcut for this. But it is still not clear to me if the Authors will research only the general time-coupling or synchronization of behaviours or the timings of the imitative behaviours only. In other parts of the paper Authors write about “imitative perception-action timings” or simply “imitation” (in the Introduction).

Reply 2.3. We thank the reviewer for pointing out this unclarity. We have indeed specifically studied the coupling (i.e., matching) of reaction times of responses (i.e., timing of actions) to stimuli. When discussing our study in a broader context, we now consistently use the terms perception-action coupling (instead of pairing) and imitation. However, in the previous version of our manuscript we erroneously used these terms at places that required a narrower specification. These terms can be viewed as main classes under which several other sub-forms fall, including spatial action mimicry, synchronization of repetitive actions, and reaction time coupling. When discussing the results of the current study, we now use the term reaction time coupling rather than the more general term perception-action coupling.

Comment 2.4. Evoking learning seems to point to this narrower interpretation, I am not sure about social bonding. Both learning and bonding are not the only reason why people would synchronize their imitative or complementary actions – the most obvious reason is cooperation in dyads and groups for which basic time-synchrony seems to be important as shown in many studies (e.g. research by Carol Fowler, Kerry Marsh, Michael Richardson, Kevin Shockley, Rick Dale or Alex Paxton). This larger context, in which imitation for learning and bonding could be presented, is ignored in the paper. It is a pity because learning takes place mostly while doing things WITH others (for which timing is also important) and not observing others and imitating the shape and timing of their moves. Perhaps such a larger context would help the reader understand better the motivations for posing the the research question: for this reviewer it was not clear, why is it important to know if our coupling of action timings is stronger because of the competence or liking of the others? Why are the two contrasted with each other, while they could well be both?

Reply.2.4 We thank the reviewer for suggesting to incorporate the literature about the cooperation in dyads and time-synchrony. We think that the research on joint action synchronization closely relates to our measurement of reaction time coupling, because both operations require the perception of spatiotemporal dynamics of actions. We also concur with the comment that learning is a social mechanism, something you do with others in joint action, and that timing is indeed crucial for successful cooperation. We have put more focus on the timing of actions and how it influences the synchronization and imitation in behavioral actions by citing the work of the suggested authors in both the introduction and discussion:

Line 67-72: It is important to note that several forms of imitation exist, including reproduction attempts of the spatial movement patterns of a person’s actions (e.g., yawning), time-coupling of (repetitive) behavior (e.g., synchronization during dancing), and becoming as ready as another person to quickly perform actions (e.g., reaction time (RT) coupling). It is the latter form that has received little scientific attention and will be of interest to the current study.

Line 87-92: Similarly, the time-coupling and synchronization of behavior during joint action also strongly depends on the social context and a person’s goal to cooperate (Abney, Paxton, Dale, & Kello, 2014; Marsh, Richardson, & Schmidt, 2009; Miles, et al., 2010; Miles, Nind, & Macrae, 2009; Paxton, Dale, & Richardson, 2016; Shockley, Santana, & Fowler, 2003). We here question to what degree the social context is relevant to the coupling of reaction times of action responses to stimuli.

Line 98-102: With the current study we address to what degree the time-coupling of responses to stimuli can be affected by a virtual agent. We do this in the context of the functional theories of imitation that we mentioned earlier. Remarkably, these theories, concerning learning, social, and cooperative views on the functional benefits of imitative perception-action coupling, have never been compared.

Line 549-567: In our experiments the likability of an agent had no effect on RT coupling. This seems at odds with the well-established imitation and joint action literature showing effects of likability of agents on behavioral resonance of actions across space and time (Abney, et al., 2014; Chartrand & Lakin, 2013; Lakin, et al., 2003; Marsh, et al., 2009; Miles, et al., 2010; Miles, et al., 2009; Paxton, et al., 2016; van Baaren, et al., 2009). The effect of likability seems robust as it even facilitates imitation and synchronization of complex or uncommon behaviors, such as facial expressions (Hofman, Bos, Schutter, & van Honk, 2012; Leighton, et al., 2010), rocking a chair (Richardson, Marsh, Isenhower, Goodman, & Schmidt, 2007), and the swinging of a pendulum during conversation (Richardson, Marsh, & Schmidt, 2005). It is important to note that such effects of likability are often investigated in physical rather than virtual, computer environments (van Schie, van Waterschoot, & Bekkering, 2008). However, research using virtual environments and RT tasks suggests that specifically the effects of the social presence of a person does not necessarily explain perception-action coupling (Dolk et al., 2011). Instead, it is the a priori knowledge about an actor’s presence that affects reaction times (Vlainic, Liepelt, Colzato, Prinz, & Hommel, 2010). Such knowledge may set the action schemes of observers, like the possibility that, as agents performed more and more correct actions, participants may have gradually strengthened their focus of attention for the actions of agents. It is thus possible that not social contexts but factors that control attention affect perception-action coupling in specifically reaction time and stimulus-response tasks.

Comment 2.5. Empirical level:
I am not sure I fully understood the design and the analyses.
The color action game was used, which had two goals: to “manipulate whether the agent played good” and “measure the degree of similarity in reaction times”, which, if I understand correctly, put the participant in a task of – sometimes – imitating the agent and sometimes suppressing the imitation and going for the right color (if the agent in the video pressed the wrong button). This occurred with various frequency for various agents (which are called functional or dysfunctional because of that).
So if someone wanted to measure the timings correlation in imitative movements, the RTs should be taken only from the correct trials of the agent and participant.

Reply 2.5. Our experiments incorporate rather complex designs but these are well understood by the reviewer.

Note that the participants made almost no errors (~5%). The reported statistical outcomes did not differ qualitatively between analyses when using only correct trials or all trials including incorrect trials. So it does not matter whether only correct trials were taken or not.

Line 278-281: Trials during which the participants responded too late were excluded from the analysis. Both incorrect trials (i.e., participants selected the wrong color) and correct trials were used for RT coupling.

Comment 2.6.Although it is not clearly written in the paper, if I understand correctly the rationale behind Experiment 3, and the lack of addressing the problem of unequal RT sample sizes (80%correct for some agents and 20% correct for the others) – this was not the case. Thus the RTs were most likely measured across all the trials (congruent – when the movements of agents and participants matched) and incongruent (when they mismatched). If this was the case, then could the results be due to the participants being more often primed for the correct action for those agents who were more often correct, while when they were more often not correct, the participants had to inhibit the whatever desire to imitate (if they had any) and perform a different action.

Reply 2.6: The RTs were indeed measured across all trials, independent of action congruency/compatibility. We did not report on differences in RT correlations across action compatibility types due to a lack of sufficient power and a different scope of our study. We believe the conditions suggested by the reviewer would be interesting to study more closely in the future, using an experimental paradigm that makes the task more difficult and included more trials to ensure enough statistical power per congruency condition. We now clarify this in the methods:

Line 285-288: Due to the scope of our research question and the relatively small number of trials, we ignored effects of stimulus-response compatibility and thus restricted the analysis to variations in the RT correlations across agent types.

Comment 2.7. In the case of agent’s movements which started before the cue for the participants (this is how -200ms could be interpreted), this additionally gave a hint to the direction, which probably facilitated the movement of the participant resulting also in fast RTs, which for the competent agents could be taken as reliable. In short: Seeing an incongruent movement the participants had to disregard or suppress the primed reaction observed in the confederate, which could take variable time depending on the strategy they used. Seeing a congruent movement, they saw it as a cue for their own.

Reply 2.7: The suggestion of the reviewer is very interesting and the described behavior has probably happened. However, the current study did not have the scope and statistical power to research action compatibility effects. The current paper only focusses on the correlations of reaction times between agents and participants.

Comment 2.8. If I am reading this wrong, and if only the “imitative” (though it is perhaps hard to call them such in a task requiring exactly the same movement) trials were taken into account then it should be explained how the problem of unequal trial numbers were dealt with. And if so – why the Exp 3 was performed (one of its aim was to eliminate the congruency bias).

Reply 2.8: We apologies for the confusion. The number of analyzed trials (correct and incorrect trials, but not late trials) did not vary across participants to a degree (see SDs per experiment) to influence the correlations. We have added these numbers to the analysis section. Furthermore, the main goal of Experiment 3 was to decouple the dependent variable (correlation of timing of actions between agents and participants) from the independent variable (functionality manipulation) within the CA task. We are not sure what the reviewer means with eliminating the congruency bias, as there was no such bias present in our design.

Comment 2.9. The task of the reader is made more difficult by the fact that the detailed instructions and full information on what the participants heard and saw is missing. How exactly was the agent introduced? As a collaborator, as an opponent? How was it explained that the video of the other person was played to the participants in the CA task? This is important as this is how participant’s action is coupled to that of the “partner” – this gives a larger setting for the task.

Reply 2.9: We thank the reviewer for pointing out that it was necessary to clarify several design and procedural aspects more clearly. We now explain the suggested aspects more thoroughly in the method section of experiment 1. We also elaborate more on how the video of the agents were introduced to the participants:

Line 164-167: Participants played these games with agents virtually, and they were led to believe, through verbal and textual explanations, that the agents were real persons whom were seated behind a computer in other rooms and that the hand movements of the agents were livestreamed to the participants.

Line 178-182: Participants played the PD and CA games with one agent per block. Before each block of 20 trials (one prisoner’s dilemma and four color action games per trial; top panel in Fig 1B), participants were shown the agent’s name and a picture of his or her face (Chicago face database; Ma, Correll, & Wittenbrink, 2015) on the screen to notify them that they would start playing with a new agent.

Line 229-230: We told the participants that this was a live video stream of the partner that they played with in the preceding PD game.

Comment 2.10. Other instructions and informations are also missing: How did the Authors ask about a person’s “functionality” (BTW I do not think labeling agents functional or dysfunctional on the basis of CA game is fortunate). Were the points in PD accumulated and visible for the participants? What was the feedback for, etc, etc….?

Reply 2.10. We clarified the questions that were asked afterwards and we added some examples of the questions to the Method section of experiment 1:

Line 271-274: We asked them to rate each agent on a functionality (i.e., How functional/well did the agent made his/her choices in the color action game?) and likability scale (i.e. how likable the agent during the prisoner’s dilemma was game) that ranged from 0 to 100 percent (fourth panel in Fig 1A; see S2 Table for all the subjective rating questions).

The points for the PD game were indeed accumulated and visible for the participants. We added this info in the method section of experiment 1 and adapted Figure 1 to match the visuals with the actual text displayed on the screen.

Line 205-208: An overview of the agent’s choice during the PD game and the accumulation of points that the participant and agent received were displayed after each PD trial (See Fig 1, top panel in A).

Other comments:
Comment 2.11. Prisoners dilemma needs often several rounds in order to establish a partner as cooperative or competitive. Here one PD game was followed by 4 CA games - could one measure (only in congruent trials) what happened on each block depending on the PD behaviour? Also, a series of cooperations or defeats should influence the judgment on cooperativity in a long run (throughout the 20 blocks). The Authors performed some split analyses (without clear motivations and hypotheses) but not this one.

Reply 2.11: We thank the reviewer for this suggestion and we actually did perform the suggested analyses earlier but these produced no noteworthy results. Considering that our manuscript is already quite extensive, we decided to not report on these analyses.

Comment 2.12. I did not understand the idea that participants might have waited when the answer was congruent. Later in the paper Authors propose that perhaps they waited at the incongruent trials (lines 404-405, this time perhaps legitimately). But it is not clear why in the first case this should have led to a greater correlation of the timings and in the second not.

Reply 2.11: Participants may wait when they have learned after several trials that a particular agent almost made no mistakes. After this discovery, participants may adopt the strategy to wait until the observed agent makes a choice before responding themselves. In this case, the reaction times of the agent will correlate with the reaction times of the participants because the participant’s reaction time directly depends on the agent’s reaction time. However, the results suggest that participants did not wait because their reaction times got shorter when playing against agents that made few mistakes. We now clarify this in the corresponding paragraph in the result section:

Line 356-358: In other words, when the participants learned after several trials that an agent almost made no mistakes, they may have waited until a functional agent responded before initiating a response themselves.

Line 367-369: …likely reflecting stronger perception-action coupling as they were following the agent’s responses rather than postponing their responses until the agent made a choice.

Comment 2.13. Authors wrote that it was possible that the participants did not believe the other has indeed been present and wasn’t an avatar – why this was not checked at the end by simply asking the participants? Also: wasn’t it highly suspicious if an agent erred on 80% of the trials in such a simple task?

Reply 2.13. The reviewer addresses a limitation of our study. We did not systematically but only occasionally ask whether the participants believed that an opponent/player was present in another room and that they played with a real person through a live-video connection. Most participants (3 out of 4) were unsure about the presence of the agents. A comment that was often put forward by the participants for why they questioned this was the logistic difficulty of setting up a live stream with four to six other people. The high percentage of errors was not specifically mentioned by participants. However, we assumed that the ecological validity of the social setting would not be a problem because the likability manipulations were very successful, meaning that participants were really annoyed by uncooperative agents. Nonetheless, we now discuss this limitation in more detail.

Line 600-615: Another limitation of the study might is related to the believability of the physical presence of the agents (Sellaro, Treccani, Rubichi, & Cubelli, 2013; Tsai, et al., 2008). At the start of the experiment, participants were led to believe they would be playing with agents via a real time video stream. During the debriefing, the experimenters occasionally asked a participant if they believed the existence and authenticity of the different agents. A majority of the participants indicated that they were not entirely sure whether they played with human agents or digital avatars via a real time video stream. Despite this uncertainty, the answers to subjective questionnaires showed that participants were conscious of the diverging behavioral profiles of the agents. They reported that the agents’ behaviors affected their feelings towards them (e.g. feelings of annoyance or even anger towards dislikable agents), making the social cooperativeness manipulation successful. Thus, despite the artificial environment used in this study, despite the possibility that some participants may have not fully believed that they were playing against a real person, and despite the unanswered question whether current results generalize to physically interactive settings, the fact that functionality but not likability affected the actions of the observer in a computerized stimulus-response task is a novel finding and the first step in comparing functional accounts of perception-action coupling.

Comment 2.14. I am at a loss why experiments 2 and 3 were conducted if the experiment one showed that the “likeability” was successfully effectuated. Also: Could the analyses be done on likeability itself and not only on the propensity to defeat and cooperate, if it was the likeability that was hypothesized to influence the timing matching and it was measured?

Reply 2.14. The likeability manipulation was successful in the sense that participants liked the cooperative agent and disliked the less cooperative agent in the prisoner’s dilemma game. However, the likeability manipulation did not result in differences in reaction time correlations. We argued that this lack of an effect of likability could potentially be explained by a too weak manipulation of likability in experiment 1 or because the likability manipulation was separated in time from the RT coupling measurement in experiment 1-2 (while the functionality manipulation was not separated). The latter could have been problematic because the effect of an uncooperative action by an agent in the PD game may not linger on to the following action trials to a degree to affect RTs. Experiment 2 was thus conducted to strengthen the likeability manipulation and experiment 3 was conducted to move the functionality manipulation forward in time (using the quiz), like the likability manipulation. Despite these improvements, only functionality but not likability had an effect on RT coupling in experiment 2 and 3.

We appreciate the reviewer’s suggestion about using the subjective ratings as the ground truth to dissociate between cooperative and uncooperative agents. Note, however, that we tested for differences in timing matching (coupling; RT correlations) across the agents. We could indeed select agents based on likability scores but this would result in exactly the same agent categories, and would be the same test as we did initially.

Comment 2.15. Experiment 3, in turn, used a strikingly different task for measuring “functionality”, tapping on completely different skills. What is the basis for claiming that competence in one task will affect the perceived competence in the other?

Reply 2.15: We thank the reviewer for this critical question. We had no scientific support for the hypothesis that the Quiz would be a successful functionality manipulation. We used our creative skills for the Quiz design and deductive skills for the hypothesis and we were eventually intrigued to see that the functionality manipulation worked so well (see Figure 2F). We also think that the Quiz is a novel, innovative aspect of our study. Regarding the competence question, it makes sense that if an agent shows reliable and useful skills in one task, that participants start paying more attention to the behavior and decision made by that agent (also see Reply 1.2).

Minor remarks:
Comment 2.16. I am confused why joint Simon task is claimed to be less interactive and involve “two person’s perform unrelated and time separated actions” (line 68)

Reply 2.16: We understand the confusion of the reviewer. We meant to say that the join Simon task does not involve a direct interaction between participants, like having a conversation. Instead, participants only look at the screen and their mere presence rather than the actual social contexts affects reaction times in this task (Tsai, Kuo, Hung, & Tzeng, 2008; Vlainic, Liepelt, Colzato, Prinz, & Hommel, 2010; Dolk et al., 2011). Nonetheless, denoting the joint Simon task as social might raise unnecessary confusion and we have therefore modified the text:

Line 73-77: Most evidence that the perception of other’s actions influences the readiness to respond and thus the reaction time of an observer comes from studies using joint action stimulus-compatibility tasks (e.g., Dolk et al., 2014). The reaction times of responses to stimulus onsets, as measured during such tasks, depend on the mere presence of another person, likely caused by the automatic interference of a mental representation of the actions of others.

Comment 2.17. In the study where cooperativeness, social bonding etc. are studied, it would be good to balance the participants group for gender or – better – included as a factor.

Reply 2.17: We have added gender as an additional random group factor to mixed linear models but it showed no effect on the RT correlations. Furthermore, we feel a bit uncomfortable with reporting gender differences, seen the context of current emancipation movements. We prefer to only report the more straightforward RM-ANOVA’s without gender as a factor, also for the sake of simplicity.

Comment 2.18. What does it mean that something is “sequentially intermixed” (line 137)

Reply 2.18. We meant to say that they alternated between each game per block of trials. We have modified this terminology as follows:

Line 162-164: In the second part, participants played two computer games (Fig 1; see S1 Video for an example of a single trial of each game). Participants alternated between the games in blocks of several trials.

Comment 2.19. The agents were 5 females and 1 male what was the reason for that?

Reply 2.19: The gender ratio was approximately matched to the participant population, mostly consisting of Dutch psychology students of Utrecht University. We have added this information to the methods:

Line 183-185: The reason for this female-male distribution was to match the participants group gender ratio, based on the psychology students in the Netherlands.

Comment 2.20. 4% of the answers were errors and 5% were late – why were they not described qualitatively? Were they congruent with the wrong button presses by the “confederates? Did they occur only on incongruent trials?

Reply 2.20: For qualitative descriptions, we would like to refer the reviewer to line 250-253. Furthermore, we have added more details regarding these trials (e.g., means and standard deviations) per experiment at the start of each Result section. Note that we did not focus on the compatibility/congruency effects. However, the percentage of incorrect and late trials did not differ across agents in any of the three experiments and we have added these findings to each result section.

Line 279-281: Trials during which the participants responded too late were excluded from the analysis. Both incorrect trials (i.e., participants selected the wrong color) and correct trials were used for RT coupling.

Comment 2.21. I have several reservations regarding the statistical analyses and reporting but they have no consequences in the face of the more significant doubts above.

Reply 2.21: We would be very happy to hear more about how we can improve the statistical analyses if the reviewer thinks this is still necessary after the current revision. Please note that any analyses related to congruency are out of scope of the current report.

Comment 2.22. In summary I do not think this work managed to show that "functionality" affects timing sychronization and have to recommend rejection.

Reply 2.22. We understand the reviewers initial opinion, which we believe was mostly caused by confusion raised by unclear terminology and several lacking methodological details. We may have also got off on the wrong foot by adding the term compatibility in the title although we were not interested in compatibility effects at all. Nonetheless, due to the reviewers’ thoughtful suggestions, we were able to solve these issues and we are confident that the paper has now reached the appropriate level of quality for publication in PLOS One.
