# Supplementary material for: Reaction time coupling in a joint stimulus-response task: A matter of functional actions or likable agents?
Source: PLoS One. 2022 Jul 12;17(7):e0271164. doi: 10.1371/journal.pone.0271164 (PMC9275686; doi:10.1371/journal.pone.0271164)
Supplement: Attachment — Submitted filename: Response to reviewers.docx [file pone.0271164.s013.docx]

**Reviewer #1, comment 1.0:** I would like to thank the authors for taking my comments and suggestions seriously. I think the edited changes make the document more understandable (for example, in the Method). The authors incorporated a wider literature review, and also provide additional acknowledgement regarding non-social explanations for the findings (with a nod to Dolk’s 2014 review article – although I am still confused by the interpretation of Dolk – see below).

Although the authors have made changes to reduce to provocative nature of the title and the text, I think further work needs to be done to acknowledge the vast research that has looked at coupling/synchronisation in all of its complexities. For example, the introduction and discussion give the impression that this study would definitely determine whether coupling (in RT contexts) has functional or social bonding benefits. For example in the Discussion “Although it is tempting to propose” with what is written following that is too strong. The results presented in this manuscript, although novel, is not convincing enough to change my view regarding the role of coupling/synchronisation in perceptual-motor tasks.

The authors introduce the multitude of benefits for synchronisation with the inclusion of additional references in this revision. I think it is enough to justify the study. However, I think the interpretation of the results should be constrained to this particular task context without language that gives the impression that the role of coupling/synchronisation is X over Y - where the literature suggests that X and Y are both important.

**Reply 1.0:** We thank the reviewer for the positive feedback. We have implemented more citations regarding coupling/synchronization to ensure that all relevant literature is now covered. We also agree with the reviewer that the literature suggests that social (Y) and non-social (X) aspects are important for coupling/synchronization to occur, despite that our study, with its specific design, shows no effects of (social) likability. We have tempered several statements in the discussion regarding social effects (see below for details).

Section comments:

Abstract:

**Comment 1.1:** “Here we employ a joint-action task” – that sentence should be rewritten to be clearer on what aspect was “joint” (the prisoner dilemma game. I would not call the color game a joint action task).

**Reply 1.1:** We have changed this to: *“…employ a visuomotor task …”* and specified the type of games in a later sentence: *“…in preceding prisoner dilemma games and quizzes, …”.*

**Comment 1.2:** “Functionality and likeability – be more specific. “Functionality = predictability that the agent will make the same response. “Likeability” – cooperation in prisoners dilemma game.

**Reply 1.2:** We clarified these constructs in the following way: *“To manipulate functionality and likability, we varied the predictability of cooperative behavior and correctness of actions of agents, respectively. Participants played against likable (cooperative), dislikable (uncooperative), functional (correct actions), and dysfunctional (incorrect actions) agents.”*

**Comment 1.3:** The last sentence “these findings suggest” include “are more likely to adopt”.

**Reply 1.3:** Corrected.

Introduction:

**Comment 1.4:** I am confused by the interpretation of the Dolk et al 2014 review article. Dolk et al also review research that demonstrate that performing a “joint” task next to a metronome or a cat can also induce these effects. How do we make sense of these results and the results by Tsao 2008? I think a couple more sentences are needed that reviews these lines of research (and anything more contemporary) to justify the use of the artificial agent setup to pursue research question.

**Reply 1.4:** We thank the reviewer for this suggestion. We implemented a more thorough review of the literature to justify the use of the artificial agent setup:

*“..using joint action stimulus-compatibility tasks (for a review, see Dolk et al., 2014). A popular perspective on such effects is that the response times to stimulus onsets, as typically measured during such tasks, depend on the mere presence of another person, likely caused by the automatic sharing of and interference between mental representations of the actions of others (for a review, see Sebanz, Bekkering, & Knoblich, 2006).”*

*“An alternative, non-social explanation for behavior coupling (and synchronization) also exists. Termed as the “Referential Coding” account of coupling behavior in joint action tasks (i.e., mostly in the joint Simon task), Dolk and colleagues (2014; 2013) suggest that an action is stored in the brain as a code (representation) consisting of multiple features, such as the speed of a response (e.g., fast or slow) and the physical changes it evokes in the environment (e.g., the clicking sound of a button press). The features of self-produced and observed actions, independent of whether these are produced by a social being, can overlap in code content. When a feature of two actions (e.g., the location of a button) becomes incongruent, interference in coding occurs, which consequentially may slow reaction times. The strongest evidence for this non-social account of behavioral coupling comes from studies showing that reaction times in joint action tasks do not depend on the social identity of co-acting agents but mostly on how participants process spatial aspects of actions of others and how such observed action representations interfere with one’s own action plans (Ferraro et al., 2012; Milanese, Iani, Sebanz, & Rubichi, 2011). Also, reaction time interference or synchronization even emerges in competitive, non-social contexts (Naber, Vaziri Pashkam, & Nakayama, 2013; Ruys & Aarts, 2010). In the light of the aforementioned social and non-social accounts, we here investigate to what degree social and non-social aspects are relevant to the coupling of reaction times of action responses to stimuli.”*

*“An interesting approach to study the importance of social versus non-social effects on RT coupling is the use of virtual agents because their behavior and the social context can be controlled well. Interestingly, virtual co-actors can even be invisible and still influence reaction times of participants in joint stimulus-compatibility tasks, though only when participants believe that the agent is a biological being rather than digital entity (Tsai, Kuo, Hung, & Tzeng, 2008). It is to be noted that the digital or virtual aspect of an agent’s presence may reduce the amount of attention for their actions and consequentially temper effects on reaction time coupling as compared with a “live” agent presence which naturally attracts more attention (Sellaro, Treccani, Rubichi, & Cubelli, 2013). Thus, besides the belief of having an intentional (virtual) partner (also see Stenzel et al., 2012), sufficient attention for the partner’s actions, either drawn bottom-up through salient events (Dolk et al., 2011; Dolk, et al., 2013) or top-down though instruction (Müller, Brass, et al., 2011; Müller, Kühn, et al., 2011), is required to modulate reaction times of the observer. Similar arguments about the importance of the focus of attention have been provided by studies on mimicry of physiological states, such as pupil size (Derksen, van Alphen, Schaap, Mathot, & Naber, 2018; Mathôt & Naber, 2018).”*

**Comment 1.4:** Line 110 “the chance to team up” – in the context of this experiment, there is no chance to “team up” during the reaction time task so how can likeability influence behavior in this context?

**Reply 1.4:** We now wrote “the chance to bond”.

Results:

**Comment 1.5:** 1.) Please add effect sizes to your tests.

**Reply 1.5:** We have added effect sizes (Cohen’s d; partial eta squared) to the statistical test results.

**Comment 1.6:** 2.) Was there an official debriefing protocol that was followed to determine whether participants believed they were interacting with a human? What % of participants were/were not convinced?

**Reply 1.6:** We thank the reviewer for this suggestion as we did not implement an official debriefing protocol. We have addressed this as a limitation of our study in the discussion. It is to be noted, however, that we observed synchronization with RTs of all agents, independent of the believe that participants were interacting with a human and independent of action functionality and likability. The degree of synchronization only varied between agents with functional and dysfunctional actions. Still, it is possible that likability may also affect synchronization when the action intentionality and believability of the actors is enhanced. As discussed in **Reply 1.4**, the amount of attention for an action is likely a mediating factor, with more believable actors drawing more attention to their actions. However, we ensured that attention was always focused on the action of the actor, for example by superimposing relevant color cues on top of the actor’s hand (for details, see Methods). As such, we think that it is unlikely that the current results were confounded by whether participants believed that they were interacting with a human.

“*One not yet considered factor that may have affected the results is the believability of the physical presence of human agents (Sellaro, et al., 2013; Tsai, et al., 2008). At the start of the experiment, participants were led to believe they would be playing with agents via a real time video stream. During the debriefing, the experimenters occasionally asked a participant if they believed the existence and authenticity of the different agents. A majority of the participants indicated that they were not entirely sure whether they played with human agents via a live real time or prerecorded video stream. Despite this uncertainty, the answers to subjective questionnaires showed that participants were conscious of the diverging behavioral profiles of the agents. They reported that the agents’ behaviors affected their feelings towards them (e.g. feelings of annoyance or even anger towards dislikable agents), making the social cooperativeness manipulation successful. Also, we ensured that full attention was paid to the actions of the agents by superimposing the color cue on top of the hand performing the action. As attention is likely the mediating factor of effects of believability of human presence (Dolk, et al., 2014; Sellaro, et al., 2013), we do not expect that the artificial context and believability explains the lacking effects of likability in the current study. Thus, despite the artificial environment used in this study, despite the possibility that some participants may have not fully believed that they were playing against a real person, and despite the unanswered question whether current results generalize to physically interactive settings, the fact that functionality but not likability affected the actions of the observer in a computerized stimulus-response task is a novel finding and the first step in comparing functional accounts of perception-action coupling.*”

Discussion:

**Comment 1.7:** Last paragraph. “Although it is tempting to propose” This last sentence needs to be reworked. The role of synchronisation is multi-faceted. I would simplify is that the role of synchronisation is for coordination of minds. This has a function role (to help with timing of actions, to be “in sync” on how to solve a task) as well as a bonding/social role (to build rapport, to empathise). I would argue that the results in this experiment, given that there is no true interactive context, are not strong enough to “tilt” the scale towards synchrony having functional over social benefits.

I have no issues regarding the results themselves, but I would advise the authors to really temper down claims of generalisation to coupling/synchronisation generally.

**Reply 1.7:** We agree with the reviewer and we have tempered down general claims by specifying that our results apply only to “virtual interactions”:

*“We are confident that, at least in virtual stimulus-response compatibility tasks and for temporal aspects of behavior, the presence of action errors is one of the main drivers of RT decoupling.”*

*“It is tempting to propose that the function of RT coupling – and perhaps also of other forms of perception-action coupling – is strongly driven by the possibility of taking advantage of learning (Hasson, et al., 2012; Heyes, 2001; Meltzoff & Marshall, 2018; Meltzoff & Moore, 1977; Piaget, 2013; Prinz, 1990) and dynamic dyadic action coordination in joint tasks (Hasson & Frith, 2016; Wiltermuth & Heath, 2009). The role of bonding, however, remains to be confirmed in future studies that may adopt the current design and incorporate more realistic, physical settings. We hope that more research will shed light on the cause of the intriguing underlying mechanisms that allow us to copy useful actions from others without too much creative, internal effort that is normally required for self-produced actions.”*

**Comment 1.8:** Article to consider:

A recent article (Wohltjen & Wheatley 2021 “Eye contact marks the rise and fall of shared attention in coversation” would be a useful resource for the authors which investigates coordination in a naturalistic context. In particular, the discussion section has a lot of good articles that might be of value related to the social and functional aspects of synchrony in human interaction.

Another article that might be useful for you (although less clear): Dotov et al 2019 “The Role of Interaction and Predictability in the Spontaneous Entrainment of Movement.” The description of the abstract seems the most relevant, but the authors investigated participants participants to synchronise to auditory signals that were either interactive or predictable.

**Reply 1.8:** We thank the reviewer for these interesting studies. Especially the studies cited by Wohltjen & Wheatley were very useful and we now cite these throughout the manuscript.

Reviewer #3: Review of PONE-D-20-38766R1, Functionality rather than likability drives reaction time coupling.

This joint-action response time study is motivated by the social synchronization literature. Shared goals and a common social context often result in coordination and synchronization in individual's movements, gestures, and communication. This article tested for influences originating from both the functionality and the likability of virtual partners. The results indicate that the participants response times became correlated with those of the virtual agents. On the other hand, social likability did not appear to influence the outcomes. The authors conclude that perception-action synchronization is more important for the SR compatibility task.

**Comment 2.1:** The authors express a bit of surprise that likability did not impact RT performance. However, I don't find it too surprising. Most of those published studies involved interpersonal interactions that evolved over some length of time (e.g., conversations, games). The synchronization measure typically emphasized events on much shorter time scales (gestures, movements, utterances). Most of these situations require adjustments just to maintain the social interaction.

**Reply 2.1:** We agree with the reviewer that in the context of existing literature, our results make sense. Indeed, studies reporting social effects on behavioral coupling and synchronization tend to in socially interactive situations. To highlight this point, we wrote:

*“This may seem at odds with the well-established imitation and joint action literature showing effects of likability of agents”*

*“It is important to note that such effects of likability are often investigated in physical rather than virtual, computer environments (van Schie, van Waterschoot, & Bekkering, 2008).”*

**Comment 2.2:** By contrast, the likability manipulation and the RT task happen on relatively fast and similar time scales. And their nature varies as a function of trial. I agree that a lack of functionality is the crucial factor given the design. However, one must be mindful that all the activities involved in going to a lab, the consent process, and the interactions with competent and engaged experimenter is a compelling social context for participants. The authors are good experimenters, and my hunch is the participant's desire to please the researchers overrode the annoyances or distortions of the agents. Replace your well trained team with a pedantic, condescending, annoying and disinterested researcher for a couple days, and I guarantee your lab's error rates will explode, and your RT's will be a hot mess.

**Reply 2.2:** The reviewer describes a very interesting hypothesis and we wished that we would have data to test this. For now we leave this hypothesis as it is and hope to be able to test it in the near future.

**Comment 2.3:** In my judgment, the manuscript meets or exceeds the 7 PLOS publication criteria. There may be some skepticism because the crux outcome constitutes a single pattern of correlation. However, it is replicated during the course of separate studies. The limitations in the discussion could be balanced a bit with more complete discussion of the synchronization hypothesis/outcome. In this regard, the authors may want to consult the literature on synchronization and fractal scaling in response time tasks. It is a better fit than the social sync literature, given the outcomes.

**Reply 2.3:** We thank the reviewer for the positive feedback. We have extended the discussion with a sentence on fractal scaling analyses (and recurrence quantification) in response time tasks:

*“Also, the complexity of sequential actions in interactive dyads have not been explored, with fractal scaling and recurrence quantification as potentially insightful analyses for how synchronization of reaction times may dynamically vary over time (Davis, Brooks, & Dixon, 2016; Fusaroli, Rączaszek-Leonardi, & Tylén, 2014; Malone, Castillo, Kloos, Holden, & Richardson, 2014; Zapata-Fonseca, Dotov, Fossion, & Froese, 2016).”*

**Comment 2.4:** While too few data points are collected in the present task to allow the same analyses to be conducted, this literature has clearly established that response time performances are rooted in synchronization principles. 1/f noise is a stochastic form of multi-scale synchronization. There are many studies on this phenomenon. Adding noise to inter-trial intervals (ITIs) of response time tasks whitens the scaling, which is symptomatic of weaker coordination. Participants entrain to sinusoidal ITI fluctuations, and express frequency detuning, and mutual entrainment in dyadic temporal estimation performance. Sequential effects in two-alternative choice response time task were successfully captured with a discrete sine-circle map of the Haken Kelso Bunz (1985) bimanual coordination model. The participants index fingers behave as coupled oscillators, and the response durations and error rates are predicted, in surprising detail, by Farey tree ratios. Limited analyses of some of the sequential effect patterns (e.g., repetition) could be executed on the data in this manuscript. In the future, the authors should include explicitly dynamic manipulations of entrainment and synchronization, and collect many more trials. It is difficult to make a compelling case for something as dynamic as synchronization with a statistical toolset that was designed for static systems (e.g., t-tests, ANOVA, correlation etc.). Fractal analyses, recurrence quantification analysis and related statistical tools are helpful in this regard. My recommendation for publication is NOT contingent on these recommendations, but they might improve the manuscript, and inspire new studies.

**Reply 2.4:** The reviewer extends on the interesting effects of inter-trial dependencies of response times. However, our design does not allow the analysis of such effects because trials were blocked in only small groups of 4 consecutive trials, interspaced with other games for the functionality and likability manipulations, rather than continuously repeated one after the other. Nonetheless, we now discuss these types of analyses in the discussion as a relevant future aim (see reply 2.3).
